# Leptin Promotes Allergic Airway Inflammation through Targeting the Unfolded Protein Response Pathway

**DOI:** 10.1038/s41598-018-27278-4

**Published:** 2018-06-11

**Authors:** Handong Zheng, Dandan Wu, Xiang Wu, Xing Zhang, Qin Zhou, Yan Luo, Xin Yang, Cameron J. Chock, Meilian Liu, Xuexian O. Yang

**Affiliations:** 1 Department of Molecular Genetics and Microbiology, University of New Mexico Health Sciences Center, Albuquerque, NM 87131 USA; 2 Department of Biochemistry and Molecular Biology, University of New Mexico Health Sciences Center, Albuquerque, NM 87131 USA; 3 College of Agronomy, Hunan Agricultural University, Changsha, Hunan 410128 China; 4 Department of Parasitology, School of Basic Medical Sciences, Xiangya School of Medicine, Central South University, Changsha, China; 5 Autophagy, Inflammation and Metabolism Center of Biomedical Research Excellence, University of New Mexico Health Sciences Center, Albuquerque, NM 87131 USA

## Abstract

Allergic asthma and obesity are major public health problems in the world. Recent Meta-analysis studies implicated a positive relationship between serum leptin, which is elevated in obese individuals, and the risk of asthma. However, it is not well understood how obesity-associated elevation of leptin increases the risk of asthma. In the current study, we have found that leptin induces the unfolded protein response factor XBP1s in an mTOR- and MAPK-dependent manner in pro-allergic TH2 cells; *in vivo*, mice fed with high fat diet had increased serum leptin as observed in human obese population and exacerbated asthmatic symptoms, associated with increased XBP1s expression in splenic CD4^+^ T cells. XBP1s is required for leptin-mediated pro-allergic TH2 cell survival and cytokine production. Our results reveal a previously unappreciated insight that obesity-associated hyperleptinemia contributes to enhanced pro-allergic lymphocyte responses through induction of XBP1s, leading to exacerbation of allergic asthma.

## Introduction

Asthma is a chronic lung disease that inflames and constricts the airways, leading to breathing difficulty. Asthma nowadays affects 300 million people worldwide and more than 25 million people (7%) including 7 million children in US (ref.^1^; CDC data, 2015). Allergic asthma is the most common type of asthma (60% of all cases), which is triggered by inhaled allergens such as pollen, dust mite, pet dander, mold and so on. Unfortunately, asthma currently is not curable and thus, could only be controlled by taking medicine as well as avoiding contact with potential environmental allergens. Moreover, severe asthmatic condition is often refractory to medical treatment and life threatening. Airway hyperreactivity (AHR), IgE-mediated sensitization, and immune cell infiltration, especially eosinophil infiltration are typically described as hallmarks of allergic airway disease^2,3^, which are majorly induced by type 2 immune response. T helper type 2 cells (TH2), an essential subset of CD4^+^ T cells, have been shown to play a key role in arousing type 2 inflammation in allergic asthma after primed and sensitized by allergens. Allergen-reactive TH2 cells express canonical type 2 cytokines IL-4, IL-5 and IL-13. Among these, IL-4 induces and maintains TH2 cells, and also contributes to IgE-producing B cell isotype switch and population expansion^4,5^. IL-5 recruits and activates eosinophils leading to eosinophilia^3^. IL-13 could directly target on airway epithelial cells for induction of AHR and mucus production^5^. In contrast, regulatory T cells, the classical negative immune regulators, produce anti-inflammatory cytokines such as TGF-β and IL-10 that block lymphocyte activation, therefore restricting excess and harmful allergic response^6^. Besides T helper cells, bronchial epithelial cells produce a wide array of cytokines under allergic pro-inflammatory condition to promote type 2 immune responses, including IL-33, IL-25 and thymic stromal lymphopoietin (TSLP)^7–11^. Group 2 innate lymphoid cells (ILC2s), a subset of recently defined innate lymphoid cells, have been shown to possess a crucial role on the development of type 2 allergic inflammation by producing type 2 cytokines, including IL-5, IL-13 and IL-9^12–14^. At the beginning, the recruitment and activation of ILC2s rely on epithelial cell-derived cytokines IL-33, IL-25 and TSLP^12^. The maintenance of ILC2s is dependent on IL-9 secreted from ILC2s and TH9 cells^15–17^. After activation, ILC2s secrete IL-13 to promote DCs migration and therefore, enhance TH2 cell priming and memory development^18,19^. Interestingly, ILC2s express MHC class II and costimulatory molecules on their cell membranes, indicating a potential antigen presenting capacity of ILC2s in mediating CD4^+^ T cell activation^20,21^. However, how dysregulation of interplays between multiple immune cells and airway mediators results in induction and aggravation of allergic asthma is unclear, and merits further study.

Besides asthma, obesity is another leading health problem worldwide and meta-analysis studies manifested that obesity is a major risk factor for development of asthma^22–24^. However, the underlying mechanism whereby obesity increases the risk of asthma has not been well established. Leptin, a classical pro-inflammatory adipokine mainly derived from adipocytes, displays significantly higher amounts in serum and visceral adipose tissue from the obese population than the non-obese population^25,26^, leading to defects in modulating balance between food intake and energy expenditure followed by multiple autonomous complications. Leptin resistance has been described to enhance parasympathetic tone, which leads to bronchoconstriction and obesity-associated asthma^27^. Dysregulation of leptin expression by adipose tissue has been shown to influence lung physiology and mechanics, and associates with asthma development^28^. In addition, hyperleptinemia was proposed to be a crucial factor leading to respiratory failure in leptin-resistant obese subjects^29^. Meta-analysis studies have further implicated a positive relationship between serum leptin and the risk of asthma^30–32^. Thus, leptin emerges as a potential mediator driving allergic asthma. However, how leptin participates in the interconnection between adipose tissue and airway inflammation and how leptin-involved defective metabolism influences lung physiology are not well understood.

Our previous studies have shown a pathogenic role of leptin in enhancing allergic responses^33^. We found leptin deficiency leads to attenuated asthma symptoms with decreased eosinophilia, and type 2 cytokine production. Leptin targets the mTOR, MAPK and STAT3 pathways in TH2 cells^33^. However, it is not fully understood how these signals lead to enhanced type 2 cytokine production. In this study, we found that leptin induced XBP1s [spliced form of X-box binding protein 1 (XBP1)], an endoplasmic reticulum (ER) stress-unfolded protein response (UPR) factor important in secretion function of both T and B cells for secretion of cytokines and antibodies, respectively^34–36^. We found XBP1s contributed to leptin functions not only in cytokine production but also in cell survival in pro-allergic TH2 cells. *In vivo*, splenic CD4^+^ T cells from high fat diet-fed mice (termed HFD mice) with plethoric leptin and exacerbated asthma symptoms, close to obese human subjects, express increased amounts of XBP1s relative to normal chow diet-fed group (termed ND mice). Taken together, our data suggest that the leptin-XBP1s axis upregulates the responsiveness of pro-allergic TH2 cells. These findings provide a novel insight into the potential of obesity-associated elevation of leptin leading to the increased risk of allergic asthma.

## Results

### Leptin induces an endoplasmic reticulum (ER) stress-UPR factor, XBP1s

**Figure 1:**
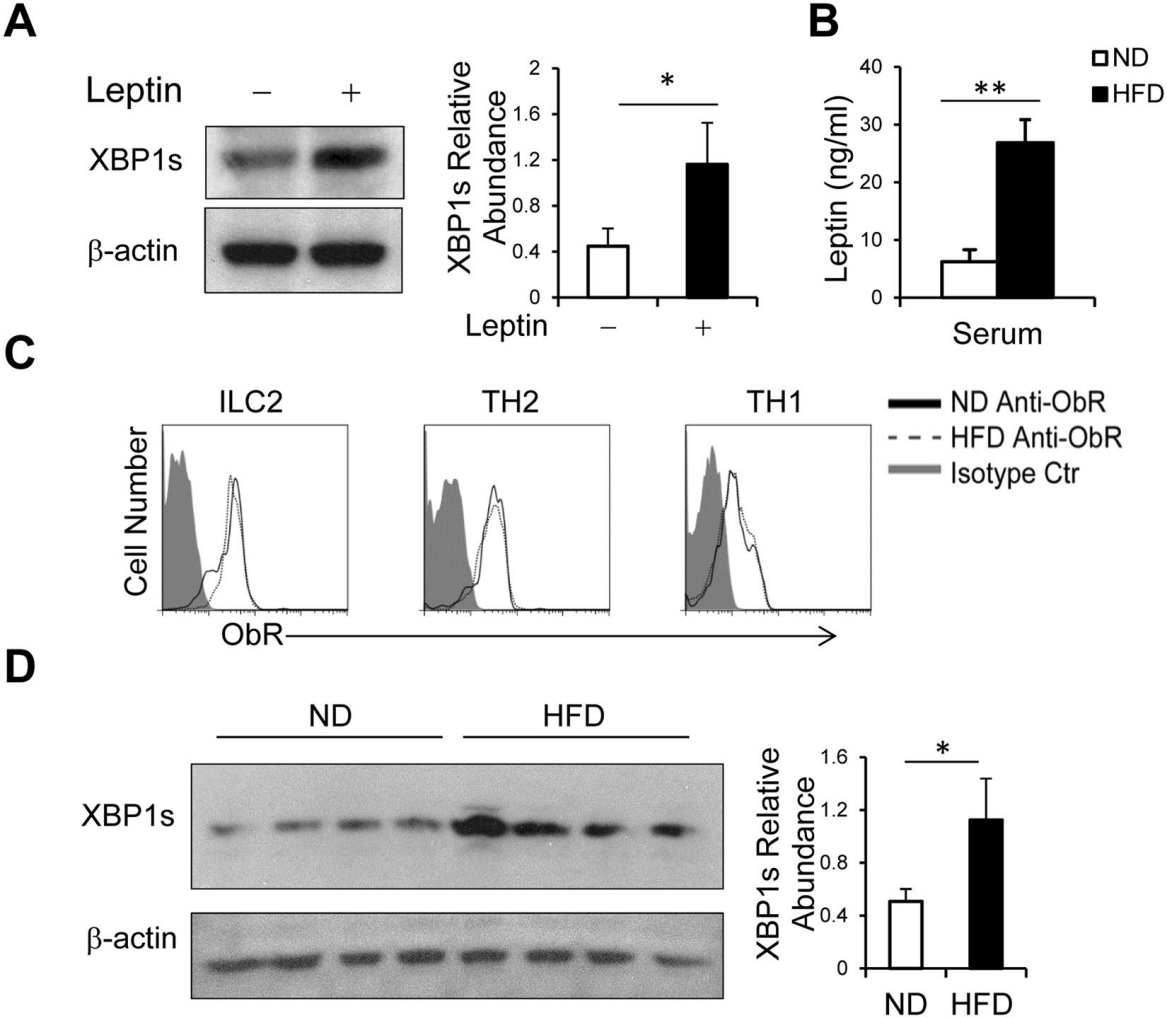
Leptin induces expression of the UPR factor XBP1s. (**A**) Immune blot of XBP1s expression in *in vitro* generated TH2 cells with or without treatment with leptin. (**B**) ELISA of leptin expression in sera of HFD and ND mice. (**C**) Flow cytometry of ObR expression on LLN ILC2, TH2 and TH1 cells from asthmatic ND and HFD mice. (**D**) Western blot of XBP1s expression in splenic CD4^+^ T cells from ND and HFD mice. (A right, D right) Quantification of XBP1s abundance was relative to β-Actin. (A right, B,D right) Values are means and SD [n = 3 (A right) or 4–6 per group (B,D right)]. Student’s t-test *p ≤ 0.05 and **p ≤ 0.005. Data represent 2 (B,C,D) or 3 (A) experiments.

We have previously found that leptin deficiency impairs type 2 immune responses and attenuates allergic airway inflammation through attenuation of pro-allergic TH2 cells and ILC2s^33^. ER stress-UPR play a pivotal role in T and B cell secretion function. To further understand how leptin-mediated signals regulate pro-allergic cytokine expression, we examined expression of the ER stress-UPR factor XBP1s in *in vitro* polarized TH2 cells in the presence or absence of leptin, and found that treatment with leptin induced XBP1s expression compared with no treatment (Fig. 1A). To test the effect of leptin on XBP1s expression *in vivo*, we generated HFD mice (body weight, 44.02 ± 2.18 g), which expressed increased levels of serum leptin compared with lean mice fed with ND mice (33.92 ± 2.77 g) (Fig. 1B), replicating human studies^37^. We asked whether HFD affected leptin receptor (ObR) expression on various types of immune cells. By using flow cytometry, we found that TH2 cells, ILC2s and TH1 cells from lung draining lymph nodes (LLNs) of HFD and ND mice all expressed comparable levels of ObR (Fig. 1C), indicating that the regulation of ObR is independent of diet or serum leptin levels. Next we examined XBP1s expression in splenic CD4^+^ T cells isolated from the HFD versus ND mice and found that HFD CD4^+^ T cells expressed elevated levels of XBP1s protein compared with ND cells (Fig. 1D). These results indicate that leptin induces XBP1s expression, which may contribute to leptin-induced hyper-responsiveness of pro-allergic TH2 cells.

### HFD-associated elevation of leptin correlated with aggravated allergic airway inflammation

**Figure 2:**
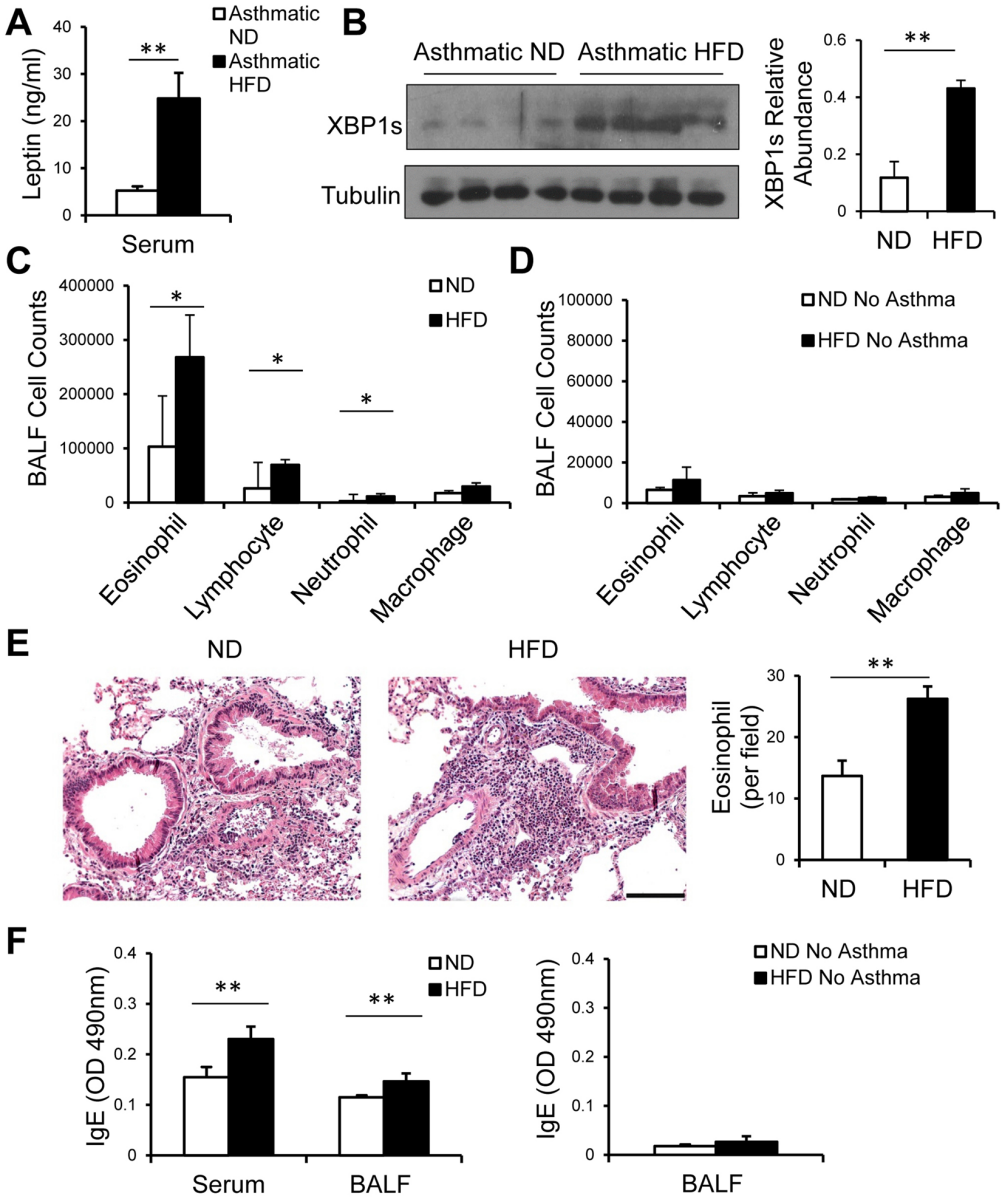
HFD exacerbates allergic airway inflammation. (**A**) ELISA of leptin expression in sera of HFD and ND mice after induction of asthma. (**B**) Western blot of XBP1s expression with Tubulin as a loading control in splenic CD4^+^ T cells from asthmatic HFD and ND mice. (**C**,**D**) BALF cellular profile in HFD and ND mice with (**C**) or without induction of asthma (**D**). (**E**) Hematoxylin-and-eosin stain of lung sections from ND and HFD mice after induction of experimental asthma. Scale bar, 100 μm. Right panel, Eosinophil counts in lung sections. Numbers shown are means per field per section (125x). (**F**) ELISA of Ova-specific IgE in Sera and BALFs from asthmatic mice (left) and in non-asthmatic control groups (right). (A,B right, C,D,E right, F) Values are means and SD. Student’s t-test, *p ≤ 0.05 and **p ≤ 0.005. Data represent 2 experiments (n = 4–6 per group).

We have observed above increases in leptin expression in HFD mice compared with their ND controls, and during allergic asthma, HFD T cells expressed higher amounts of XBP1s. We then tested allergic inflammation in the HFD and ND asthmatic mice as previously described^33,38^. We found that during allergic responses, HFD mice had increased levels of leptin in serum relative to ND mice (Fig. 2A) and elevated expression of XBP1s in splenic CD4^+^ T cells (Fig. 2B), similar as the steady state (Fig. 1B,D). HFD mice displayed increased infiltrates of eosinophils, lymphocytes and neutrophils in bronchoalveolar lavage fluids (BALFs) compared with ND mice (Fig. 2C). Whereas, PBS challenged HFD and ND mice displayed basal level of BALF immune cell infiltration (Fig. 2D). To evaluate lung inflammation, we stained the lung sections from HFD and ND mice with hematoxylin and eosin. In compliance with BALFs immune cell infiltration, we observed that HFD lungs exhibited increased mononuclear cell infiltration in the peribronchovascular spaces that contained increased numbers of eosinophils (Fig. 2E). In addition, papain and Ova challenge elicited IgE responses in both HFD and ND mice; Sera and BALFs of HFD mice contained increased levels of Ova-specific IgE compared with ND mice (Fig. 2F, left). IgE expression in PBS challenged HFD and ND mice were only at basal levels (Fig. 2F, right).

**Figure 3:**
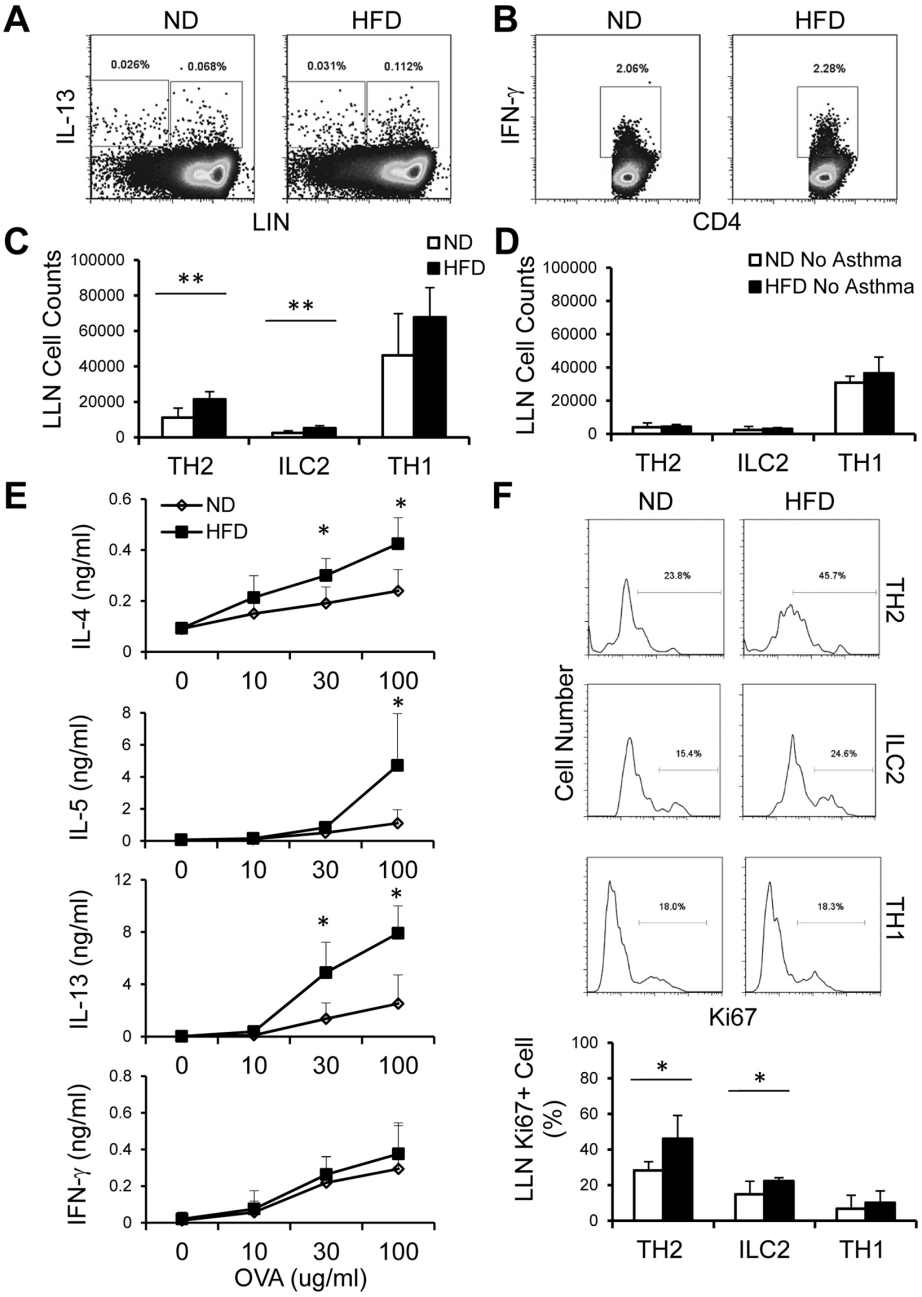
HFD leads to increased type 2 lymphocyte responses. (**A**,**B**) Intracellular stain of TH2 (LIN^+^CD4^+^IL-13^+^), ILC2s (LIN^−^CD4^−^IL-13^+^) (**A**) and TH1 cells (**B**) in LLN cells from asthmatic ND and HFD mice. (**C**,**D**) Quantification of TH2, ILC2 and TH1 cells in asthmatic LLNs (**C**) and non-asthmatic LLNs (**D**). (**E**) ELISA of cytokine expression by asthmatic LLN cells after *ex vivo* recall with Ova at indicated concentrations. (**F**) Intracellular stain of Ki67 in LLN TH2 cells, ILC2s and TH1 cells in asthmatic LLN cells. (C,D,E,F bottom) Values are means and SD. Student’s t-test, *p ≤ 0.05 and **p ≤ 0.005. Data represent 2 experiments (n = 4–6 per group).

Type 2 immune responses manifest one of the essential hallmarks of allergic asthma. Therefore, we profiled type 2 lymphoid TH2 cells and ILC2s, as well as TH1 cells in asthmatic HFD mice relative to their ND controls, and found that lung associated draining lymph nodes (LLNs) of HFD mice contained increased frequencies and numbers of TH2 cells and ILC2s but the frequencies and numbers of TH1 cells are comparable with ND mice (Fig. 3A–C). In contrast with asthmatic groups, PBS challenged HFD and ND mice had only a few TH2 cells and ILC2s in LLNs (Fig. 3D); interestingly, both PBS challenged HFD and ND mice contained slightly fewer TH1 cells in LLNs compared with the asthmatic groups (Fig. 3D). Upon *ex vivo* recall with Ova, HFD LLN cells expressed higher amounts of TH2 cytokines, IL-4, IL-5 and IL-13 and comparable amounts of TH1 cytokine IFN-γ relative to the ND group (Fig. 3E), whereas LLN cells from PBS-challenged HFD and ND mice only expressed basal amounts of these cytokines (data not shown).

Above results showed that HFD mice contained increased TH2 and ILC2 frequencies and expressed more Ova-specific type 2 cytokines versus ND mice. We next asked whether HFD promotes proliferation of these immune cell populations during allergic responses. To address this, we measured the expression of Ki67, a cell proliferation-associated nucleic protein that marks cell at active phases (G1, S, G2 and M) but not the resting phase (G0), in TH2 cells, ILC2s and TH1 cells from the asthmatic mice. We observed that the frequencies of Ki67^+^ cells were greater in HFD TH2 cells and ILC2s than corresponding ND cells. In contrast, there was no apparent distinction of Ki67^+^ TH1 cells between HFD and ND mice, which was due to the dominant type 2 immune environment eliciting by a protease type adjuvant papain (Fig. 3F). Taken together, these findings suggest that HFD induces elevated expression of leptin that induces the UPR factor XBP1s and likely renders TH2 cells and ILC2s but not TH1 cells more responsive to antigen stimulation during a type 2 challenge, which subsequently activate and recruit more immune cells, including eosinophils, to the airway and lung, leading to exacerbation of allergic airway inflammation, consistent with our previous observations on leptin deficient mice^33^.

### Leptin-XBP1 axis is required for TH2 cell cytokine expression

**Figure 4:**
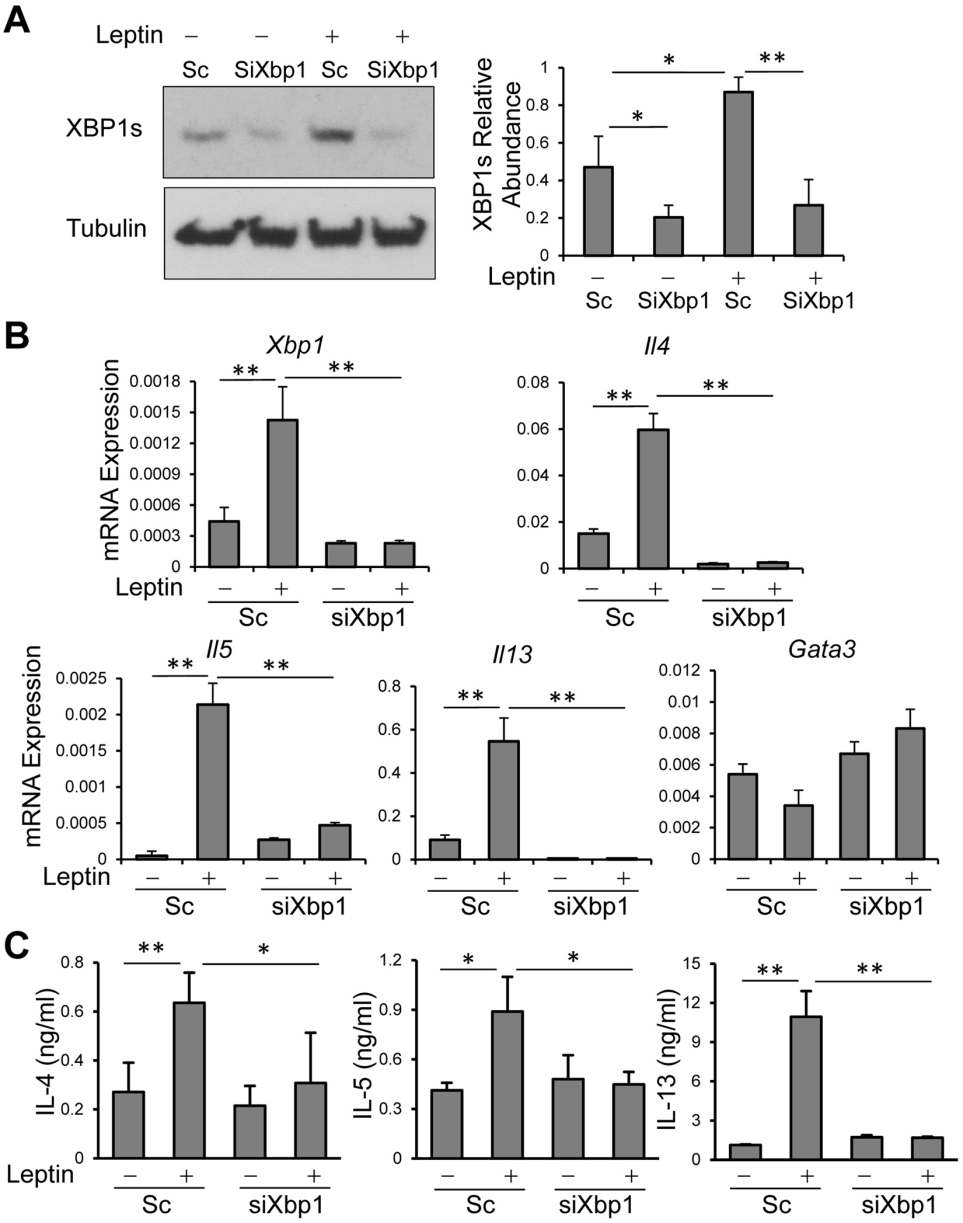
XBP1s mediates leptin-induced TH2 cytokine production. (**A**) Western blot of XBP1s abundances with Tubulin as a loading control in *in vitro* differentiated TH2 cells following transfection of siXbp1 or scramble siRNA (Sc) for indicated times in the presence of leptin for 40 h. (**B**) RT-qPCR of mRNA expression in TH2 cells treated as (**A**). mRNA abundances were normalized to an internal housekeeping gene *Actb*. (**C**) ELISA of cytokine expression in TH2 cells treated as (**A**). (A right, B,C) Values are means and SD (n = 3–4 biological replicates per group). Student’s t-test, *p ≤ 0.05, and **p ≤ 0.005. Data represent 2 (B,C) or 3 experiments (A).

Since leptin induces XBP1s expression in TH2 cells, we asked whether leptin regulates TH2 cell cytokine secretion via the XBP1s pathway. To explore this, we performed siRNA-mediated *Xbp1* gene silencing in differentiated TH2 cells. *In vitro* differentiated TH2 cells were transfected with siXbp1 or scramble siRNA with or without addition of leptin. We found that expression of XBP1s protein and mRNA was significantly upregulated by leptin treatment and leptin-induced XBP1 expression was downregulated by siXbp1 relative to scramble siRNA treatment (Fig. 4A,B), indicating a successful *Xbp1* gene silencing. In the absence of leptin, siXbp1 led to downregulation of XBP1s protein and a strong trend of decreasing its mRNA (Fig. 4A,B). We next examined whether *Xbp1* gene silencing affects the induction of cytokine expression in TH2 cells by leptin treatment and found that leptin-induced elevations of TH2 type cytokines (IL-4, IL-5 and IL-13 at both mRNA and protein levels) were reversed by *Xbp1* gene silencing (Fig. 4B,C), whereas neither leptin treatment nor *Xbp1* knockdown altered the expression of *Gata3* mRNA (encoding GATA3, the master transcription factor of TH2 cells). These data indicate that the leptin-XBP1s axis is required for TH2 cell cytokine expressions.

### Leptin-XBP1s pathway protects TH2 cells from activation induced cell death

**Figure 5:**
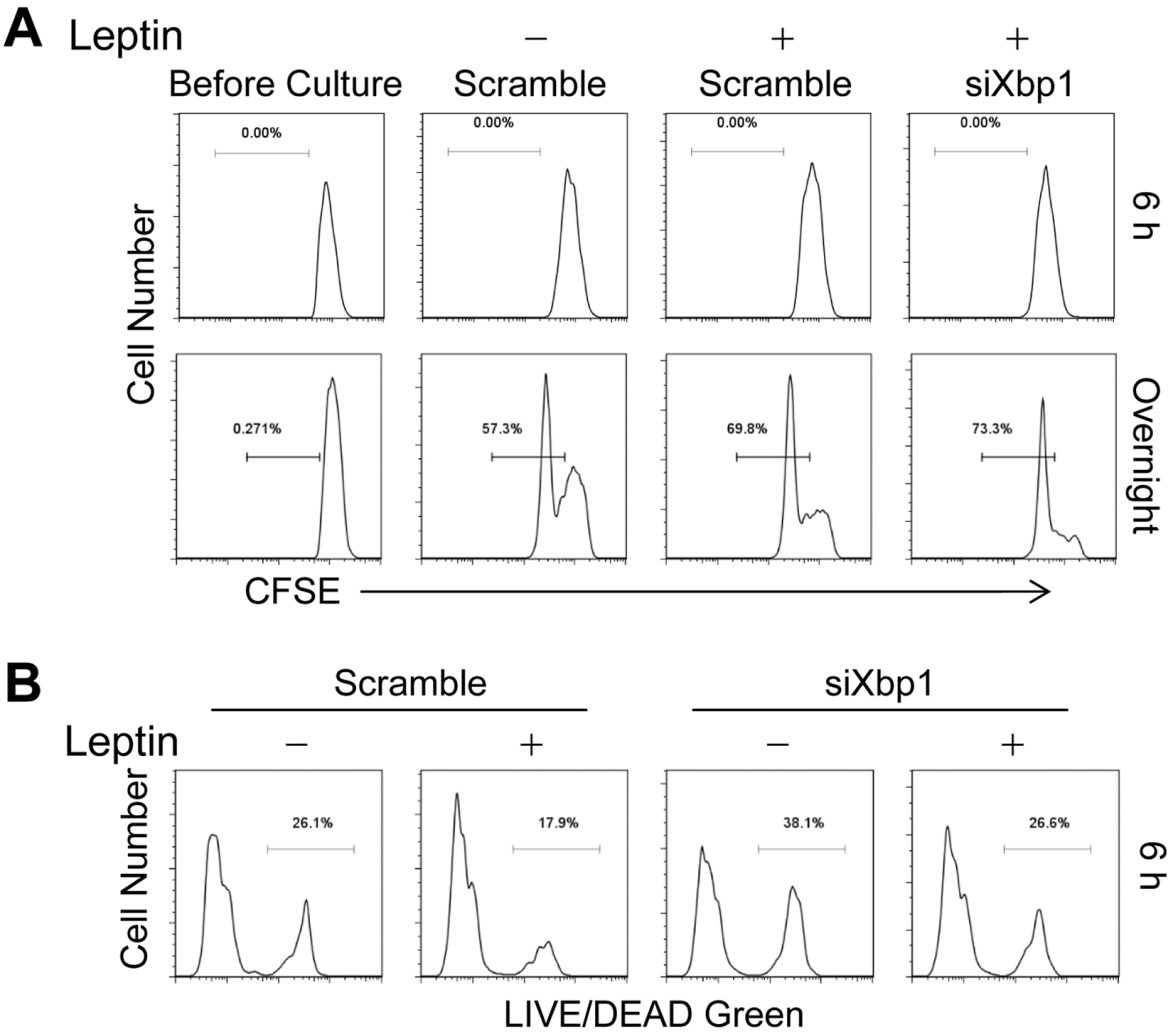
XBP1s is required for leptin to protect TH2 cells from activation induced cell death but not to promote their proliferation. (**A**) Flow cytometry of CFSE dilution in TH2 cells after 6 h or overnight restimulation in the presence or absence of leptin with siXbp1 or scramble siRNA treatment. (**B**) Flow cytometry of activation induced cell death in TH2 cells after 6 h restimulation as in (**A**). Data represent 2 experiments.

Although XBP1s is known to enhance cell secretion through regulation of ER function^35,36^, it is not clear whether XBP1s also contributes to the other effects of leptin. We assessed the effect of XBP1 on proliferation of siXbp1 or scramble siRNA transfected TH2 cells for 6 h or overnight culture by CFSE dilution. We found that leptin increased TH2 cell proliferation after overnight but not 6-h culture; addition of *Xbp1* gene silencing did not alter the effect of leptin on proliferation (Fig. 5A). In addition to proliferation, we measured activation induced cell death in *in vitro* differentiated TH2 cells by LIVE/DEAD Green stain and found that leptin-mediated protection on cell death was abolished by *Xbp1* gene silencing, indicating an essential role of XBP1 in controlling cell survival (Fig. 5B). Therefore, XBP1s is required for leptin mediated cell survival but not proliferation of pro-allergic TH2 cells.

### Leptin induces XBP1s expression in a MEK- and mTOR-dependent manner

**Figure 6:**
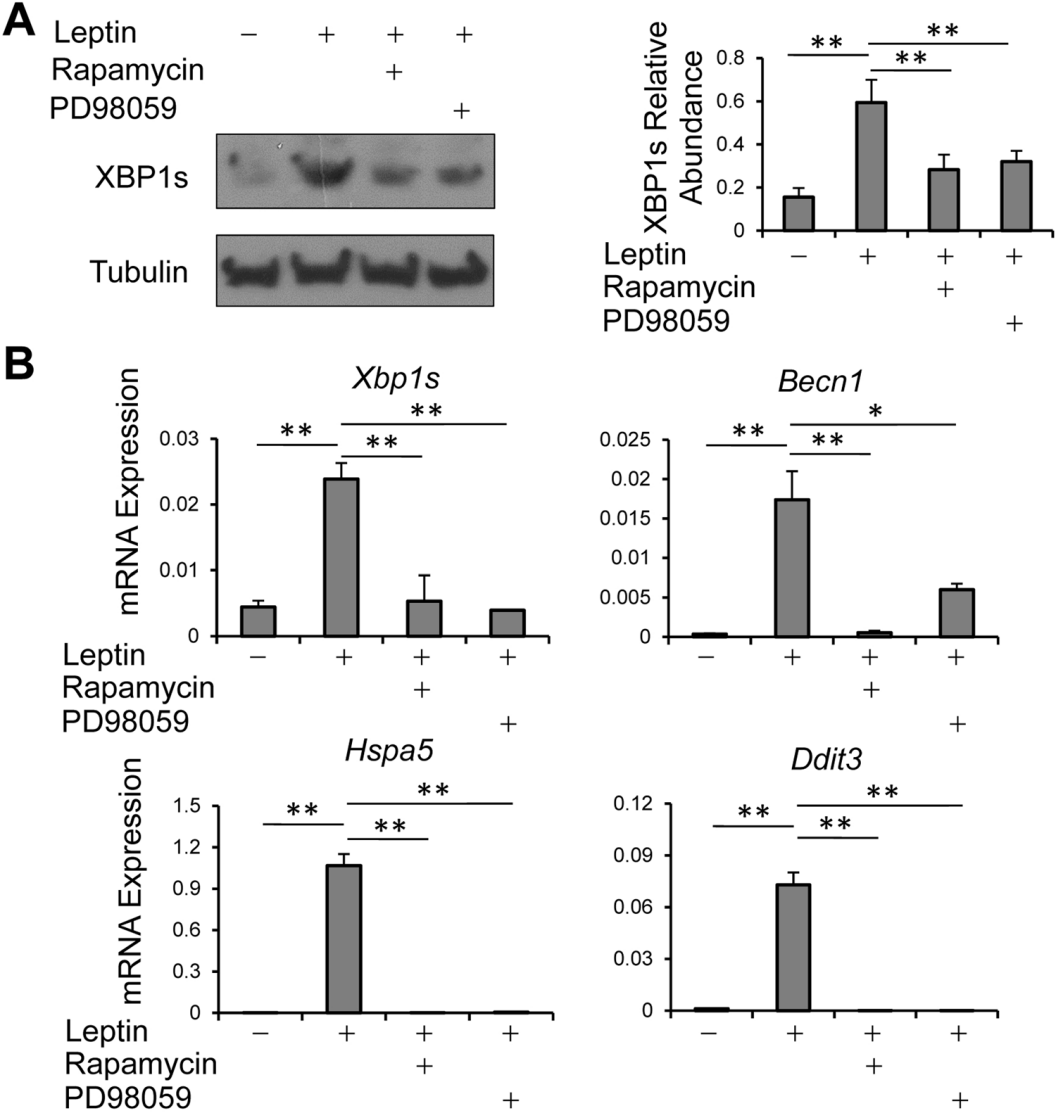
Leptin induces XBP1s expression through the mTOR and MEK pathways. (**A**) Western blot of XBP1s expression in TH2 cells following treatment with or without PD98059 (20 μM) or rapamycin (200 nM) in the presence or absence of leptin for 4 h. XBP1s abundances were normalized to Tubulin. (**B**) RT-qPCR of mRNA expression of *Xbp1s* and XBP1s downstream genes. mRNA abundances were normalized to *Actb*. (A right,B) Values are means and SD (n = 3 biological replicates per group). Student’s t-test, *p ≤ 0.05 and **p ≤ 0.005. Data represent 2 (B) or 3 (A) experiments.

Our above results indicate leptin functions through induction of XBP1s expression (Figs 1A, 4 and 5B). Leptin is known to activate the mTOR and MAPK pathways in TH2 cells^33^. We next asked whether these leptin signals are able to induce XBP1s expression. We found that leptin-induced XBP1s expression could be blocked by addition of either mTOR inhibitor rapamycin or MEK inhibitor PD98059 (Fig. 6A), both of which can block induction of TH2 cell cytokine expression by leptin^33^. XBP1s is known to transactivate genes encoding factors promoting ER function and autophagy^39,40^. We therefore assessed whether leptin signaling affects the expression of XBP1s downstream factors and found that leptin induces *Hspa5* (encoding UPR factor BiP), *Ddit3* (encoding UPR factor CHOP) and *Becn1* (encoding autophagy factor Beclin1), whereas treatment with both mTOR inhibitor rapamycin and MEK inhibitor PD98059 greatly diminished the effects of leptin on induction of these UPR and autophagy factors (Fig. 6B). Thus, leptin regulates XBP1s expression through activation of the mTOR and MEK signal cascades in TH2 cells and induces UPR and autophagy factors which is likely through the induction of XBP1s.

### Leptin induces XBP1s expression through activation of IRE1 but not ATF6

**Figure 7:**
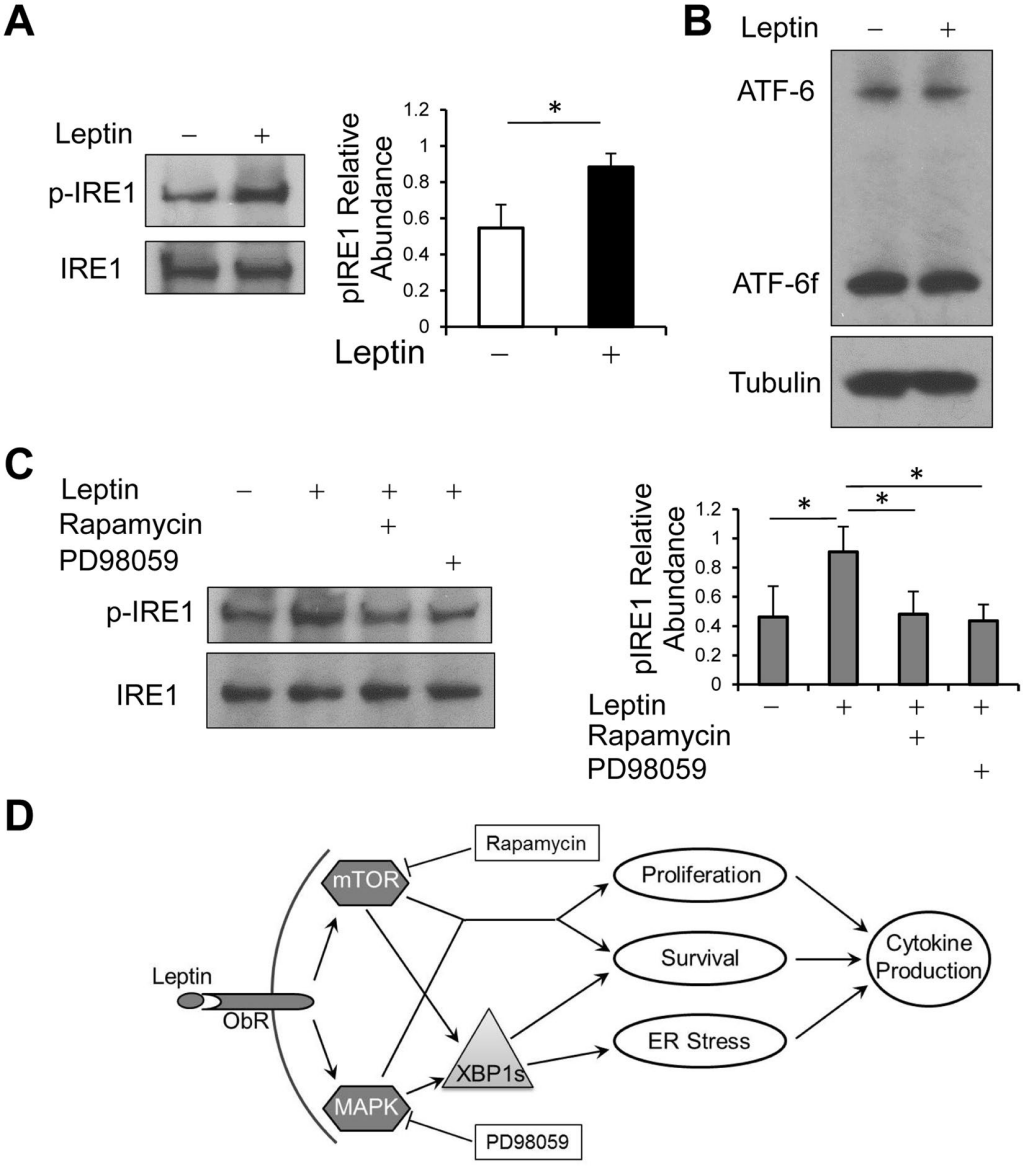
Leptin induces IRE1 but not ATF6 activation through the mTOR and MEK pathways. (**A**) Western blot of p-IRE1 and total IRE1 expression in TH2 cells treated with or without leptin. (**B**) Western blot of ATF6 expression in TH2 cells treated with or without leptin. ATF6f, cleaved active form of ATF6. (**C**) Western blot of p-IRE1 and total IRE1 expression in TH2 cells following treatment with or without PD98059 (20 μM) or rapamycin (200 nM) in the presence or absence of leptin for 8 h. (A right, C right) p-IRE1 abundances were normalized to total IRE1. Values are means and SD (n = 3 biological replicates per group). Student’s t-test, *p ≤ 0.05. Data represent 2 (C) or 3 (A,B) experiments. (D) Outline of the effects of leptin-mTOR/MAPK-Xbp1 axis in pro-allergic TH2 cells.

After activation, TH2 cells are known to mount massive protein synthesis leads to ER stress-associated UPR, in which activation of IRE1 and/or ATF6 results in increased expression of *Xbp1s* mRNA and XBP1s protein^35^. To understand how leptin signaling induces XBP1s expression, we examined IRE1 and ATF6 activation by western blot. We found that addition of leptin induces IRE1 phosphorylation (Fig. 7A), whereas it did not alter ATF6 cleavage (Fig. 7B), suggesting that leptin induces XPB1s expression through activation of IRE1 rather than ATF6. We next assessed whether the mTOR and MEK pathways downstream of leptin activate IRE1 and found both mTOR inhibitor rapamycin and MEK inhibitor PD98059 could block leptin induced phosphorylation of IRE1 (Fig. 7C). Therefore, leptin activates the mTOR and MEK pathways that subsequently activate IRE1, leading to increasing XBP1s expression and finally enhancing pro-allergic TH2 cell function.

Taken together, our results demonstrate that obesity-associated elevation of leptin may result in activation of the mTOR/MEK (upstream of MAPK)-IRE1-XBP1 axis in pro-allergic lymphocytes that promote cell survival and cytokine production, therefore exacerbating allergic airway disease (outlined in Fig. 7D).

## Discussion

Obesity was identified as a major risk factor in development of allergic asthma by meta-analysis^22–24^. Many studies have shown that leptin, an adipokine highly elevated in the obese population, represents a paramount role on affecting lung physiology and mechanics and thus, regulating respiratory function, which is correlated with obesity-associated asthma^27–29^. Furthermore, a positive relationship between serum leptin and risk of asthma has been revealed by meta-analysis studies^30–32^. However, as a potential mediator driving allergic asthma, leptin is still in lack of being understood on how it participates in defective metabolism-involved lung pathology. Several studies showed that leptin promotes TH1 responses both *in vivo* and *in vitro*^41–43^, whereas, whether leptin regulates pro-allergic type 2 responses is not yet elucidated. Batra *et al*. showed leptin promoted TH2 cell development and aggravated TH2-mediated colitis, however, controversial results *in vitro* showing high concentration of leptin (1 μg ml^−1^) reduced TH2 cell frequency in repeated polarization cultures have been described in the same study^43^. This might be explained by over-dose leptin raised toxicity that could overwhelm its physiological effects. In a human childhood study, the obese asthmatic group had increased plasma IL-4 and IFN-γ, correalted with higher plasma leptin compared with control gorup^42^. ILC2s, another essential component in type 2 immune responses and allergic asthma, produce type 2 cytokines^12–14^, promote TH2 cell differentiation and memory responses in allergic asthma^18–21^. Furthermore, activated TH2 cells produce IL-2 that promotes ILC2s development. This reciprocal regulation between TH2 cells and ILC2s suggests a feed-forward loop in allergic responses. Our current studies showed that HFD mice with plethoric leptin had increased TH2 and ILC2 proliferation and type 2 cytokine production compared with ND group, which contributes to the exacerbated asthma symptoms.

Leptin modulates immune cell function through activating multiple downstream signaling pathways including JAK2-STAT3, MAPK and PI3K-AKT^44,45^. For instance, leptin enhances TH1/TH17 cell survival through activating ERK1/2 and AKT-mTOR pathways^46^. In consistency with these studies, we have shown that in TH2 cells, leptin also activates STAT3, MEK-MAPK and AKT-mTOR signaling pathways, which contribute to leptin-mediated cell proliferation, survival and cytokine production^33^. It has not been well understood how these leptin signals regulate type 2 cell cytokine production. Upon extracellular stimulation, lymphocytes produce massive amounts of effector cytokines. Accumulation of unfolded (or misfolded) proteins leads to ER stress that activates the UPR pathways^35^. During UPR, ATF6 is cleaved into an active ATF6 fragment (ATF6f) transcription factor, and induces transcription of several genes, including *Xbp1*; subsequently, endoribonuclease IRE1 undergoes phosphorylation and phosphorylated IRE1 excises a 26-nucleotide fragment from unspliced *Xbp1* (*Xbp1u*) mRNA and forms spliced *Xbp1* (*Xbp1s*) mRNA. XBP1s protein transactivates transcription of many genes that are crucial for secretory function through increasing ER capacity and promoting autophagy^40,47^. These pathways together allow a cell to resolve the endogenous stress of unfolded proteins and maintain intracellular homeostasis. Therefore, XBP1s plays a central role in UPR and cell secretion function. For example, in human necrotizing enterocolitis, *XBP1* splicing levels correlate with the severity of mucosal damage that is associated with increased mucosal expression of pro-inflammatory cytokines, IL-6 and IL-8^48^. In our study, we have observed that leptin induces XBP1s expression in TH2 cells and after knock-down of *Xbp1*, the effect of leptin on TH2 cell cytokine production is diminished (Fig. 4B,C). Previous studies have shown that *Xbp1* mRNA splicing can be induced by MAPK signaling in liver cells or PI3K-AKT-mTOR signaling in innate immune cells^49,50^. We have also found that leptin induces XBP1s expression dependent of both MEK-MAPK and mTOR signaling pathways, which leads to activation of IRE1 but not ATF6 (Fig. 7), and XBP1s is required for the anti-apoptotic effects of leptin in TH2 cells (Fig. 5B). In summary, our results suggest leptin as a key risk factor in the development of allergic asthma in obese subjects through induction of the UPR factor XBP1s that promotes survival of pro-allergic lymphocytes and their cytokine expression. These findings may suggest a novel therapeutic approach for treatment of obesity associated allergic asthma.

## Materials and Methods

### Animals

Six-week-old C57BL/6 mice were fed with either a normal chow diet (ND) or HFD (45 kcal% fat, D12451; Research Diets Inc., New Brunswick, NJ, USA) for 15 weeks. All mice were housed in the specific pathogen-free animal facility at the University of New Mexico Health Sciences Center. All animal experiments were performed with protocols approved by the Institutional Animal Care and Use Committee of the University of New Mexico Health Sciences Center. All methods were performed in accordance with the relevant guidelines and regulations.

### Induction of allergic asthma

Age and sex-matched C57BL/6 HFD and ND mice were immunized intranasally with 25 µg papain and 50 µg chicken Ovalbumin (Ova) (or treated with PBS as no asthma controls) for three times on D0, D1 and D14. On D15, Sera, BALFs, LLNs, and left upper lung lobes were collected for analysis of infiltrates and immune responses as described before^33,38^.

### *In vitro* TH2 cells differentiation

CD4^+^CD25^−^CD62L^+^ naïve T cells were sorted from C57BL/6 WT mice and differentiated in a TH2-polarizing condition (5 μg ml^−1^ anti-IFN-γ and 10 ng ml^−1^ IL-4) using plate-bound α-CD3/α-CD28 and low serum (3–5% FBS)-containing RPMI medium. Afterwards, the resulting cells were re-stimulated in serum free medium (OpTmizer^TM^ CTS^TM^ T-Cell Expansion SFM, Life Technologies) for intracellular cytokine expression, apoptosis and proliferation assay, and siRNA silencing assay in the presence or absence of leptin (200 ng ml^−1^) as indicated.

### Gene silencing

*In vitro* differentiated WT TH2 cells were starved 24 h in serum free medium on D4. On D5, the TH2 cells were transfected with *Xbp1* or scramble siRNA (Santa Cruz Biotechnology, Inc.) and incubated on an anti-CD3 coated plate for 6 h, and afterwards the siRNA transfected TH2 cells were subjected to different treatments as indicated, and were used for cytokine expression, proliferation, cell death and immunoblot assays.

### Immunoblot

Splenic CD4^+^ T cells isolated from the asthmatic HFD and ND mice were lysed immediately, and subjected for immunoblot of XBP1s. *In vitro* differentiated TH2 cells were starved for 24 h in serum free medium and then be subjected to different treatments as indicated and cell lysates were prepared for immunoblot analysis of XBP1s. Immunoblot antibodies were anti-XBP1 (M186, sc-7160, Santa Cruz Biotechnology), anti-α-Tubulin (eBioP4D1, eBioscience), anti-β-Actin (BA3R, Thermo Fisher Scientific), anti-IRE1 (B-12, sc-390960, Santa Cruz Biotechnology), anti-phospho-IRE1 (ab48187, Abcam), and anti-ATF6 (F-7, sc-166659, Santa Cruz Biotechnology).

### ELISA

Mouse Leptin ELISA kit (#90030, Crystal Chem) was utilized for leptin measurement following the manufacturer’s instruction. To measure Ova-specific IgE, plate-bound Ova (100 μg ml^−1^) were used as capture and anti-mouse IgE (23G3, eBioscience) as detection antibody. LLN cells (4 × 10^6^ cells ml^−1^) from the asthmatic HFD and ND mice were recalled with various concentrations of Ova for 3 days and the supernatants were collected for measurement of cytokine expression by ELISA using a standard protocol. For *in vitro* differentiated TH2 cells, the cells were starved for 24 h and transfected with siXbp1 or scramble siRNA. The resulting cells were washed and treated with or without leptin (200 ng ml^−1^) for 6 h, and finally the supernatants were collected and used for measuring cytokines expression by ELISA.

### RT-quantitative (q) PCR

Gene mRNA expression was determined by RT-qPCR as described previously^38,51^. Data were normalized to an *Actb* reference gene. The primers were: *Actb*, forward, 5′-GACGGCCAGGTCATCACTATTG, reverse, 5′-AGGAAGGCTGGAAAAGAGACC; *Gata3*, forward, 5′-AGGGACATCCTGCGCGAACTGT, reverse, 5′-CATCTTCCGGTTTCGGGTCTGG; *Il4*, forward, 5′-CACCACAGAGAGTGAGCTCGTC; reverse, 5′-ACTTGGACTCATTCATGGTGCA; *Il5*, forward, 5′-ACACAGCTGTCCGCTCACCGAG, reverse, 5′-TCACACCAAGGAACTCTTGCAG; *Il13*, forward, 5′-TGGGTCCTGTAGATGGCATTGC, reverse, 5′-GGGCTTCATGGCGCTCTGGGTG; *Xbp1s*, forward, 5′-CTGAGTCCGCAGCAGGT, reverse, 5′-TAATGGCTTCCAGCTTGGCT; *Becn1*, forward, 5′-CTGAGGCGGAGAGATTGGAC, reverse, 5′-CACTCCACAGGAACACTGGG; *Hspa5*, forward, 5′-AAGCGCCTCATCGGACGCAC, reverse, 5′-AACAACTGCATGGGTAACCT; *Ddit3*, forward, 5′-ATCTTGAGCCTAACACGTCG, reverse, 5′-TGGACACCGTCTCCAAGGTG.

### Proliferation assay

*In vitro* TH2 cell proliferation was assessed by carboxyfluorescein succinimidyl ester (CFSE, C34570, ThermoFisher Scientific) dilution. 1 d after siXbp1 or scramble siRNA transfection, TH2 cells were labeled with CFSE and re-stimulated with plate-bound anti-CD3 in serum free medium with or without leptin (200 ng ml^−1^) for 6 h or overnight incubation. For *in-vivo* proliferation, single-cell suspensions of LLNs from the asthmatic mice were prepared and restimulated with PMA, Ionomycin in the presence of Golgi blocker, and Ki67 expression was measured by intracellular stain.

### Cell death assay

One day after siXbp1 or scramble siRNA transfection, TH2 cells were re-stimulated with plate-bound anti-CD3 in serum free medium with or without leptin (200 ng ml^−1^) for 6 h. Afterwards the cells were collected and stained with LIVE/DEAE Green (LIVE/DEAD^®^ Fixable Dead Cell Stain Kit, Invitrogen), reactive to free amines both in the interior and on the cell surface, for assessment of activation induced cell death.

### Flow cytometry antibodies

CD3e (145-2C11), CD4 (GK1.5), CD5 (53-7.3), B220 (RA3-6B2), CD11b (M1/70), CD11c (N418), Gr-1 (RB6-8C5), Ter119 (TER-119), IgE (23G3), IL-13 (eBio13A), IFNγ (XMG1.2) and Ki67 (SolA15) were purchased from eBioscience; IL-4 (11B11) and IL-5 (TRFK5) were from BioLegend; ObR (AF497) and anti-goat IgG (NL002) were from RnD Systems; and goat IgG isotype control (sc-3887) was from Santa Cruz. Lineage (LIN) markers include CD3, CD5, B220, CD11b, CD11c, Gr-1, Ter119 and IgE.

### Statistical analysis

The statistical significance of differences between groups was calculated with the unpaired Student’s *t* test. *P* values of 0.05 or less were considered significant.

### Data Availability

All data generated or analyzed during this study are included in this published article.
